# Recall of advertisements after various lapses of time

**DOI:** 10.1007/s00426-020-01408-y

**Published:** 2020-09-17

**Authors:** Donald Laming

**Affiliations:** Department of Psychology, University of Cambridge, Downing Street, Cambridge, CB2 3EB UK

## Abstract

The recall of 90 magazine advertisements, consisting of a Brand, a Picture and a Slogan, was tested by presenting one of these components as cue and asking for the other two. The advertisements were tested in groups of ten, each group requiring a sequence of 30 test trials (10 advertisements × 3 cues). Backwards analysis of the sequences of responses—that is, starting with the last trial and comparing it successively with the responses on earlier trials—identified many responses as repetitions of previous errors, replicating many of the findings in Laming, D. On the recall of errors in recall*. *JSMC Brain Science, 2019, 3: 21, https://www.researchgate.net/publication/333448328_On_The_Recall_Of_Errors_In_Recall. Nine different groups of advertisements were tested after various lapses of time up to 4 months. A comparison is made between the (short term) probability of repeating a previous error as a function of lag within a single test sequence and the (longer term) probability of retrieving an advertisement as a function of elapsed time. Both these empirical relationships can be characterised by reciprocal functions, but they are otherwise quite different. Extrapolation of long-term accessibility to short intervals suggests recall in the short term could be much better than it is; extrapolation of short-term accessibility to longer intervals poses the contrary problem. Even though the only relevant variable in this comparison appears to be lapse of time, there is a question whether this comparison between short and long term is truly like with like.

**Electronic supplementary material:**

The online version of this article (10.1007/s00426-020-01408-y) contains supplementary material, which is available to authorized users.

## Introduction

**Fig. 1:**
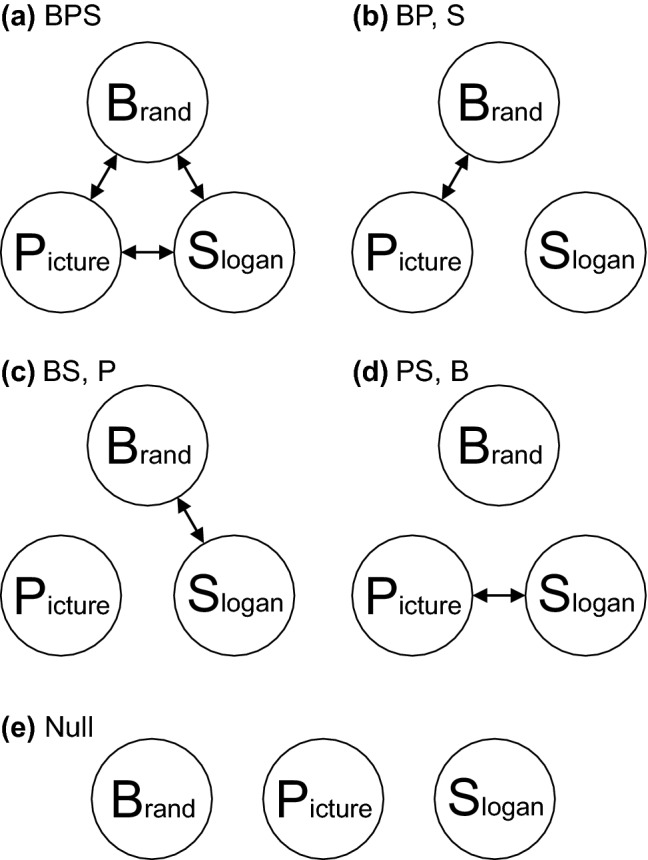
The fragmentation hypothesis for stimuli comprised of Brand, Picture and Slogan

Jones (1976) proposed the Fragmentation Hypothesis, which says, simply, that a stimulus fragments in memory. An advertisement consisting of a Brand name (B), a Picture (P) and a Slogan (S) might fragment in any of five identifiably different ways, illustrated in Fig. 1. If all three components are contained in a single fragment (BPS), then any component as cue will elicit recall of the other two. If, however, a fragment links only brand and picture (BP, S), then brand as cue will elicit recall of picture and picture as cue will give brand, but slogan is not accessible from either, nor will it retrieve anything when presented as cue. Likewise (BS, P) and (PS, B). Finally, a Null fragment yields only guesses.

The critical test of this hypothesis consists of cueing each stimulus by each of its components in turn (Jones, 1978), but separated, of course, by tests of other stimuli, and then assembling the responses to the three cues addressing each stimulus in a table (Table 1). Depending on the fragmentation of the stimulus in memory (Fig. 1), a particular pattern of responses should be correct. For example, if Brand and Picture are contained in the same fragment, but not Slogan (i.e. BP, S above), Brand as cue will retrieve the correct picture and Picture as cue the correct brand, but Slogan will be missing. Five such patterns are possible – except that the pattern in Table 1 might be augmented by a lucky guess.

**Table 1:** Responses to the three cues addressing a common stimulus

Cue/Response	Brand	Picture	Slogan
Brand	–	√	*e*
Picture	√	–	*e*
Slogan	*e*	*e*	–

Lansdale and Laming (1995) reported such an experiment, testing memory for nine colour slides of a billiards table with a coloured ball somewhere between the centre pockets (nine different colours, but not red), a distinctive pattern of eight red balls somewhere on the further half of the table (nine different patterns) and a white object on the left hand edge of the table (nine different objects). Participants were presented with one of the components from one of the stimuli and asked to recall the other two. They were asked to guess if they could not recall. There were 27 test trials of each presentation set of nine stimuli and successive trials presented a different attribute (colour, pattern or object) as cue addressing a different stimulus. While nearly half of the assembled matrices of responses to a common stimulus conformed exactly to the fragmentation hypothesis (that is, without any augmentation from lucky guesses), most of the ‘lucky guesses’ in the other half of the data turned out to be repetitions, complete or in part, of response combinations on previous test trials.

This finding led to a re-analysis of the data *ab initio*. Instead of asking of each response combination whether it was correct, Laming (2019) asked where had it come from, because the prevalence of repetitions of combinations of responses from some previous trial meant that most of those combinations must have been recorded in memory. Suppose, for example, the first recall of a stimulus is completely correct and the second cueing accesses the record of that first recall, rather than the original stimulus; this generates a spurious correlation between successive cueings of the kind implied by Fig. 1. The pattern of data that emerges persuasively suggests the fragmentation hypothesis, without that hypothesis having any relevance to memory. In this way an investigation that began with a plausible hypothesis about the structure of memories turned into an empirical exploration of recall in the short term. There are, of course, very many studies of recall over short periods of time. What was different in Lansdale and Laming (1995) is that participants had no instruction about remembering their previous responses—nothing at all was said—it just happened naturally.

This paper reports another experiment of similar design using advertisements consisting of a Brand name (B), a Picture (P) and a Slogan (S), culled from glossy magazines. It was a student project conducted during the academic year 1983/1984, with, of course, the resources available at that time.^1^ It was intended to discover, first, whether ‘everyday stimuli’ would fragment in the same way as purposefully constructed stimuli and, second, to pose this question: If, in course of time, a stimulus is forgotten, how does that come about? If the fragmentation hypothesis truly represents the state of nature, then forgetting must subsist either in fragments in memory breaking up or simply disappearing. Joensen, Gaskell & Horner (2019) have very recently posed this same question, whether a complex stimulus (a triple associate in their experiments) is forgotten all-or-none or by progressive loss of individual components. The experiment was designed to identify the fragment representations of the original stimuli after various periods of time. Nine sets of advertisements were presented for retention at the same point in time with different sets tested after different intervals ranging from 20 min to 4 months.

Anticipating the results, repetition of response combinations on preceding test trials again generates a pattern of data that persuasively suggests the fragmentation hypothesis, without that hypothesis representing any structures in memory. So the question put ceases to be relevant. Instead there is a comparison between the decreasing frequency of repetitions with lag within the one sequence of test trials and the similar decrease of accessibility over much longer delays, 20 min to 4 months, comparing the different product groups of advertisements.

## The experiment

### Stimuli

The stimuli were 90 advertisements culled from glossy magazines. Each advert consisted of a brand name (B), a picture (P) of the product and a slogan (S). They were divided into nine product groups (cosmetics, fashion, food and drink, furniture, technology, holidays, jewellery, perfumes and shoes) with ten advertisements in each group. Each product group was tested in a separate session, lest the mixing of, say, perfumes and food in the same test provided additional information which brand name, picture and slogan went together.

### Procedure

The stimuli were presented on slides in a Kodak carousel projector at 15 s intervals. The slides were ordered such that every ninth slide came from the same product group, with the different product groups cycled in a fixed order. The presentation of the stimuli began about 7 pm on a Friday evening. The participants viewed the slides as a group and then proceeded directly to the first test. There followed supper in the laboratory, with two further tests that same evening. The participants returned the following morning (Saturday, 10 am) for a further test and, thereafter, different product groups were tested at intervals as set out in Table 2. Except for the test on Saturday morning, all subsequent tests began at 6 pm. Retention intervals are measured from the mid-point of the presentation set to the mid-point of the test trials.

**Table 2:** Schedule of testing sessions in the recall of advertisements

Product group	No. of participants	Interval before testing
Perfumes	30	20 min
Holidays	28	1 h
Cosmetics	24	2 h
Food and drink	23	14.6 h (i.e., the following day)
Shoes	22	3 days
Fashion	21	1 week
Technology	14	2 weeks
Furniture	18	9 weeks (i.e., the next term)
Jewellery	16	16 weeks

### Testing

Each product group was tested with a series of 30 cues on slides, each slide showing one component from one of the stimuli. A picture cue showed the picture from the original advertisement with the brand and slogan blanked out. Brand names and slogans were typed in Times 12 pt, the brand names in capitals, and photographed against a black ground. The slides were ordered such that each stimulus was tested every 10 trials and successive slides always cued different attributes. Participants were given booklets and asked to write on each page, first the cue and then the two attributes paired with it in the stimulus.^2^ To assist, a complete set of brand names, slogans and pictures was projected on a screen. (The purpose of the experiment was to test recall of the associations between Brand, Picture and Slogan [cf. Fig. 1]; there was no expectation that participants would remember Brand or Slogan exactly.) The pictures were labelled A,…,J and the brand names and slogans were ordered alphabetically to obscure any association between them. Participants were instructed to select a guess from this screen if they could not remember. They were tested as a group, with 30 s allowed for responding to each cue. There were a small number of failures to write down any answer at all.

### Preliminary training

To explain the nature of the stimuli and the method of testing, participants were shown an example of a stimulus (a girl reaching for a bottle of Martell brandy with the slogan “Look no further”); a practice set of six advertisements for cars; and an explanation of how memory would be tested, with reference to an answer booklet, a display of the brand names, slogans and pictures of the six cars, and slides presenting the three cues for the advertisement for Martell brandy. Participants were then tested on the six car advertisements, using the procedure described above for the main experiment. This preliminary training preceded the presentation of the 90 advertisements for the main experiment.

### Participants

The participants were 30 undergraduate friends of the two students, who carried out this research project as part of their third-year course in psychology at the University of Cambridge (Paveley 1984; Wheatley 1984; personal communications). It is inevitable in such an experiment that participants will not attend all the testing sessions. The number tested decreased from 30 to a minimum of 14 over four months (see Table 1) and only 11 presented themselves for every test. The participants were tested for colour blindness in advance of the experiment using the Ishihara test. They were paid £10 for their participation.

### Terminology

‘Brand’, ‘Picture’ and ‘Slogan’ are attributes of the stimuli; a particular instance of an attribute will be called a component or, where appropriate, a response. Each product group was tested with a series of 30 *test trials,* on which the different stimuli were cued in turn, three times each. The combination of cue and two responses on a test trial is an *answer*, except that it will sometimes be necessary to distinguish answers that are copies of the answer on the immediately preceding trial, *answer (lag 0)* or, simply, *Ans0*, from the rest. The individual components of an answer are the *cue* and (two) *responses*.

**Fig. 2:**
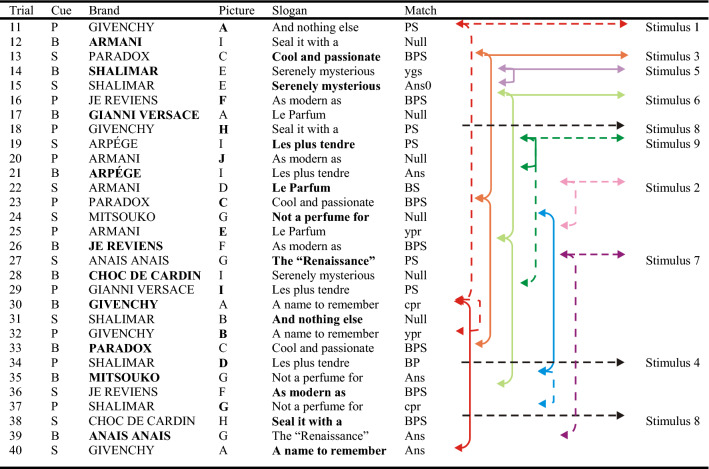
Sample data from one presentation set by one participant. Trials 1–10 are the stimuli (not shown); Trials 11–40 are the test trials. Columns 3–5 give the combination of cue and responses actually observed; the cue is additionally highlighted in bold. The arrows link each recall with its presumed source; a continuous line indicates a complete repetition, a broken line a pair match only. The nature of the match is listed in Column 6 (BPS = completely correct; BP, BS, PS = one response correct; Ans = complete recall of a previous erroneous trial; cpr = one response and the cue matching; ypr = a pair of responses, neither matching the cue; ygs = a pair of responses matching a wrong stimulus). ‘Null’ indicates two independent guesses with no identifiable source

The analysis begins by examining the repetition of previous recalls. A BPS match (links in blue in Fig. 2) indicates an answer that can be sourced, either directly (Trial 38) or via previous correct recalls (Trials 34 and 37), to the stimulus. ‘BP’,‘BS’ and ‘PS’ indicate pair matches that can likewise be sourced to a stimulus (Trials 34, 22 and 18 in Fig. 2, shown in pale blue) and the links are now shown dashed. Where one answer exactly matches the cue and two responses on some previous test trial (but not a correct recall of a stimulus), I speak of the recall of an *answer* (‘Ans’ or ‘Ans0’, green links in Fig. 2,). The presumed source of such a recall might itself be the recall of a previous error (Trials 35 and 40) or might be a complete guess (Trial 35; its source on Trial 24 has itself no identifiable source). If only one response (and the cue) match the answer on some previous trial, that is a *cued pair* (‘cpr’, Trials 30 and 37). In the case that both responses, but not the cue, match some previous answer, I speak of a *yoked pair* (‘ypr’, Trials 25 and 32). Finally, a *yoked guess* is a pair of responses that match some stimulus other than the correct one (‘ygs’, Trial 14). The analyses that follow repeatedly compare these 10 different categories of recall.

### Results

The analysis of the experiment below follows the backwards pattern in Laming (2019); that is, instead of looking only at the correlations between the three cueings of each stimulus, the analysis works backwards through each set of test trials, examining the correlations between each combination of cue and two responses and all preceding such combinations, including the original stimuli. This divides recalls into those that were sourced from within the present test series (these recalls should be unaffected by the delay since presentation of the stimuli) and recalls which necessarily access the original stimuli, distant in time.

I present two different analyses of the data:

1. An analysis of repetitions of previous errors, subdivided into
2. Incidence;
3. Conditional proportions of repetitions; and
4. Lag-recall curves.

This first analysis examines all of the data from all of the participants and is, therefore, not representative of the decreases in recall over time.

2. Comparisons of responding after different lapses of time by those 11 participants who attended all nine test sessions.

I emphasise that none of these analyses are concerned with testing the fragmentation hypothesis—only with the probable source of each response.

### Repetitions of previous errors

This first analysis seeks to establish that certain categories of error must have been recorded in memory. The participants viewed the stimuli with specific instructions to remember them, and it is taken for granted that the stimuli were indeed recorded in memory. It then becomes essential to distinguish carefully between recalls of stimuli and recalls of recalls of stimuli, on the one hand, and repetitions of errors on the other, lest the frequencies of repetition of errors be artefactually inflated with recalls of stimuli. While this might seem obvious, the second analysis below will proceed from a different standpoint.

For each test trial *n* (*n* = 11,…, 40), the analysis works backwards from trial *n*-1 to 11 and then through the stimulus set, looking for the best match to the cue and two responses. A previous answer or stimulus that matches all three attributes is always deemed a more probable source than a match of only two. Given two matches of three (or two), the most recent (smallest lag) is preferred over the more remote. So, the analysis looks first for a match to all three attributes. This may be the stimulus addressed at trial *n*, or a previous recall of that stimulus or it may be some other previous answer, of necessity containing an error. Answers that copy the answer on the immediately preceding trial (*Ans*0—an answer that might be still ‘in mind’ at the time) are distinguished from other repetitions of complete answers. An important question is whether such repetitions are merely apparent, due to chance, or whether they indicate a true recall from memory.

The probability of a match occurring by chance on trial *n*, not this particular match, but any match, depends on the number of previous answers that could be matched by a suitable choice of responses. Irrespective of whether an answer is actually recalled, and provided only there is no complete recall of the stimulus, the analysis counts the number *x* of different previous answers containing the cue presented on trial *n*. That number varies from trial to trial. There is also some number of combinations of two responses (99, because the stimulus addressed must be excluded from the calculation) that might have been output at trial *n*. The probability of matching a previous answer (any previous answer) by chance is therefore *x*/99. The occurrence of such a match is a Bernoulli variable with variance (1 − *x*/99)(*x*/99). The sum of such variables over the totality of trials is a generalised binomial, with mean equal to the sum of the probabilities and variance to the sum of the individual variances. This is compared with the number of answers actually recalled. The calculation is explained in great detail in Lansdale and Laming (1995, pp. 44–50). It turns out that the sum of the Bernoulli probabilities is small in relation to the number of previous errors that are repeated, so that most such repetitions must have been true recalls from memory.

If the answer on trial *n* is not a complete repetition of any previous answer, the analysis looks for combinations of two matching attributes. Such a combination may consist of the cue and one matching attribute (a *cued pair*) or the two attributes excluding the cue (a *yoked pair*).^3^ An answer containing a single correct response is now excluded from the calculation to preclude the number of matches being inflated by partial recalls of stimuli. If the most recent matching pair is a cued pair, or if there is no matching pair, the probability of a cued pair is calculated, along the lines set out above for an answer; except that additional combinations of cue and responses must now be excluded from the calculation because, if they occurred, they would be classified as the recall of an answer. Likewise, if the most recent matching pair is a yoked pair, or if there is no matching pair, the probability of a yoked pair is calculated. The Bernoulli probabilities and variances are summed to provide a generalised binomial variable for comparison with the total number of cued/yoked pairs recalled. The analysis of yoked pairs extends to the stimuli, where a match is separately classified as a *yoked guess*. It was discovered in Laming (2019) that yoked pairs and yoked guesses did not differ, either in assigned confidence or in the distributions of latencies. However, the distinction is important in the present study because yoked pairs are retrieved from recent test trials, while yoked guesses are retrievals from the original stimuli, which may have been presented up to four months previously. These additional calculations are also explained in great detail in Lansdale and Laming (1995, pp. 44–50).

Table 3 records the incidences of the different categories of repeated errors. ‘Total trials’ in Column 3 is the number of test trials, conditional on the cue, on which a repetition of the designated category might have been observed. These numbers relate to a total of 1960 test trials with each attribute as cue, from which double-correct answers (and, for pair matches, single–correct and repeated answers) have been deleted. Moreover, an erroneous recall cannot be repeated until it has itself been uttered, so that for test trials early in the sequence of 30 there are no previous errors that could be repeated. In the light of these restrictions, the proportions actually recorded relative to the numbers of trials on which each kind of repetition might have been observed (Answers at lag 0: 50.7%; Answers at lag > 0: 23.8%; Cued pairs: 23.9%; Yoked pairs: 17.4%; Yoked guesses: 8.6%) are considerable. These results are comparable to those obtained from a re-analysis of the data from Lansdale and Laming (1995: resp. 58.6%, 16.1%, 21.7%, 20.0% and 11.0%; see Laming, 2019).

**Table 3:** Incidences of different categories of repeated errors in the recall of magazine advertisements, with a statistical assessment of each category

Cue attribute	Response attribute(s)	Total trials	Number observed	95% confidence interval	Proportion	Chance expectation	Chi-square	Significance
Lag 0 answer fragments								
B	PS	90	44		0.489	0.000	0.000^†^	0.000
P	BS	89	37		0.416	0.000	0.000^†^	0.000
S	BP	107	56		0.523	0.000	0.000^†^	0.000
Lag > 0 answer fragments								
B	PS	581	172	2.444, 13.379	0.296	7.9118	3459.72	0.000
P	BS	531	146	2.132, 12.721	0.275	7.4265	2631.3	0.000
S	BP	609	177	2.923, 14.334	0.291	8.6286	3345.53	0.000
Cued pairs								
B	P	304	73	29.184, 52.087	0.240	40.6352	30.6844	0.000
B	S	322	37	30.588, 53.981	0.115	42.2842	0.7841	0.376
P	B	294	65	28.448, 51.091	0.221	39.7694	19.0774	0.000
P	S	273	80	25.114, 46.665	0.293	35.8895	64.3765	0.000
S	B	339	44	33.598, 57.889	0.130	45.7437	0.0792	0.778
S	P	345	105	33.998, 58.393	0.304	46.1954	89.2889	0.000
Yoked pairs								
B	PS	639	116	44.679, 72.857	0.182	58.7678	63.3878	0.000
P	BS	503	62	38.112, 64.326	0.123	51.2192	2.5988	0.107
S	BP	657	132	52.759, 82.807	0.201	67.7829	70.1813	0.000
Yoked guesses								
B	PS	639	63	32.142, 57.346	0.099	44.7439	0.005	0.005
P	BS	568	42	31.194, 56.019	0.074	43.6063	0.800	0.800
S	BP	649	54	30.731, 55.521	0.083	43.126	0.086	0.086

^†^Normal deviate from binomial test with probability 1/99

For every sub-category except four the significance is beyond dispute and most of those repetitions must have been true recalls from memory. The four exceptions are recalls of BS pairs, either to one of the attributes as cue or as a yoked pair to P as cue and also BP pairs retrieved as a yoked guess from the stimulus. The evidence relating to yoked guesses is, however, weak; aggregating the data for all three categories of cue, *χ*^2^ (*N* = 1856) = 6.238 with 1 d.f., *p* = 0.013. This is to be expected given the lapse of time between presentation of the stimuli and test.

BP and PS pairs are common, both as cued pairs and as yoked pairs, but Brand and Slogan do not cohere well; that is, the picture plays a pivotal role in the memory of these advertisements. Participants readily attach a brand or a slogan to a picture, but do not equally link brand and slogan together by themselves. In Lansdale and Laming (1995) it was colour and object that cohered—very plausibly because those two attributes had names, whereas the patterns did not.

### Conditional proportions of repetitions

**Fig. 3:**
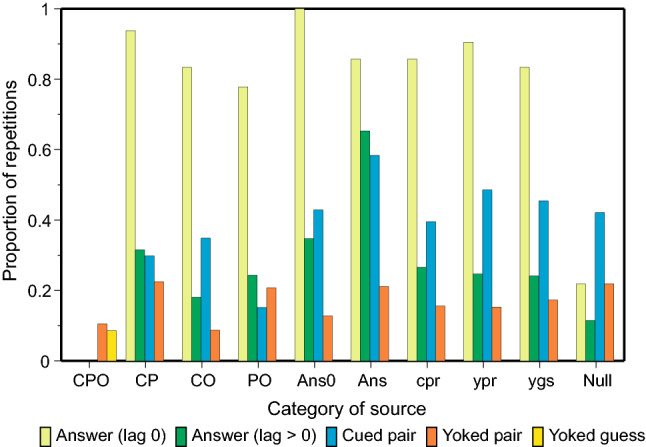
Proportions of repeated errors conditional on the nature of the source

Many repetitions are repetitions of previous repetitions. Figure 3 presents the probability of each category of repetition conditional on the presumed source. For each trial counted in ‘Total trials’ in Table 2 (col. 3) there are one or more prior trials that could have been retrieved to generate the corresponding category of repetition. The category of the source is taken from the most recent of those prior trials. The number of instances of each source serves as the denominator for the probability of repetition in Fig. 3.

**Fig. 4:**
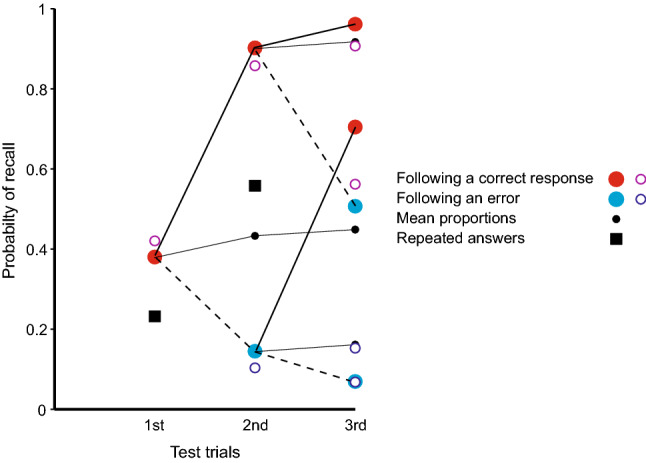
Proportions of completely correct (BPS) answers as a function of ordinal number of test trial and outcomes on preceding tests. The corresponding proportions for repeated answers are shown for comparison. The small black circles represent the mean proportions correct on the second and third trials; they are linked to the preceding data points to emphasise that the process is a martingale. The open circles are predictions from a model in Appendix A in Laming (2019).

The proportions of repetitions simply reflect a continuing incidence of repetitions as trials proceed, except for two significant features. First, if the trial cue on trial *n* + 1 is also one of the responses on the immediately preceding trial (*n*), repetition of that preceding trial (*Ans*0) is highly probable (av. 0.835), except in the case that the preceding trial is Null (two unrelated guesses with no identifiable source; 0.218). Second, the average proportion of repetitions for answers (lag > 0) is 0.244, but increases to 0.653 (see Fig. 4 below) when the source is itself an (identical) answer. Complete (erroneous) answers tend especially to be repeated.

The repetition of erroneous answers applies equally to correct recalls. A completely correct recall at first test installs an additional copy of the stimulus in memory to support an increased likelihood of a correct recall at the next test trial. Figure 4 shows the proportions of completely correct (BPS) answers following each sequence of preceding correct and incorrect test trials addressing that stimulus. The proportion correct on first test is 0.380 and, if subsequent tests depended solely on independent access to the stimulus, the proportion of three correct BPS answers would be 0.055. The proportion of three correct BPS answers in sequence is 0.330. It is plain from Fig. 4 that a correct (BPS) answer greatly increases the conditional probability of a correct answer on the next test.

There is no feedback following any of the tests in Fig. 4, no further sight of the stimulus, so that the sequence of successive recalls should be a martingale (see Laming, 2005).^4^ This means that, while the probabilities following particular sequences of recalls diverge, the expectation remains unchanged. To emphasise this property, the small black circles show the means at the second and third trials; they are linked to the preceding data points representing the proportions correct on the preceding trial. There is a slight increase in the average proportions recalled correctly (cp. Izawa, 1971, p. 201). At the same time the probabilities attaching to the different sequences of correct and wrong recalls diverge (as is obvious in Fig. 4). Continued long enough, those proportions would diverge to either 0 or 1 (cp. Izawa, 1969).

The open circles in Fig. 4 are predictions from a model in Appendix A of Laming (2019). There are three test trials for each stimulus spaced at intervals of ten within the trial sequence; the combinations of responses on those test trials have different accessibilities (probabilities of retrieval). The probability of retrieving the original stimulus, *a*_s,_ is assumed constant, but thereafter *a*_1_ is the accessibility of the responses on the previous test trial (the first trial at the second test, but the second trial at the third test) and, at the third test only, *a*_2_ is the accessibility of the first test. The model assumes the data to be homogenous when the frequencies are aggregated over nine different sets of stimuli, tested after very different lapses of time and with very different levels of correct recalls. The parameter estimates are: *â*_s_ = 0.420 for the stimulus, *â*_1_ = 0.754 for the preceding trial and *â*_2_ = 0.345. The parameter *â*_1_ is greater than *â*_2_ because it refers to a more recent trial (lag 9 instead of lag 19) and *â*_s_ is smaller, because of the lapse of time between presentation of the stimuli and test. The black squares in Fig. 4 show the corresponding proportions for repeated answers. At first test they show the proportion of answers (lag > 0) sourced from pair and Null sources (0.209) and, at second test, the proportion of answers sourced from previous answers (0.653).

To sum up: Repeated errors are often sourced from previous repetitions. The relation between source and repetition is of no apparent significance except for repetitions of the immediately preceding trial and for repeated answers. The same process applies to correct recalls, where it generates a martingale; the expected unconditional proportion of correct recalls does not change, but individual (conditional) sequences diverge.

### Lag-recall curves

**Fig. 5:**
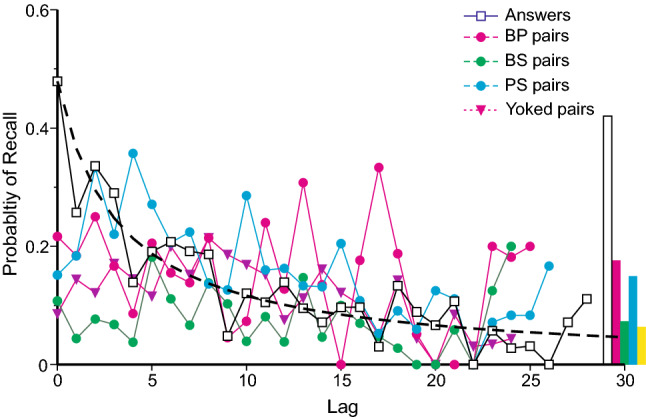
Lag-recall curves for answers, cued pairs and yoked pairs. The histogram bars at the right hand end of the abscissa are the proportions of, respectively, complete correct (BPS) recalls, correct BP, BS and PS pair recalls and yoked guesses. The black dashed curve is a reciprocal, agreeing with the proportion of answer recalls at lag 0 and approximating the variation of answer repetitions thereafter

Each recall of an answer, a cued pair or a yoked pair may be sourced from any preceding test trial, and Fig. 5 presents the probabilities that the trial at lag *l* is the source for each of these kinds of error. Recalls of previous answers and cued pairs display conventional recency, decreasing from 0.5 for the immediately preceding answer to zero at a lag of 29. This aligns the present experiment with free recall (see esp. Tan and Ward, 2000). Note that the number of answers that might be recalled decreases progressively as lag increases, and the lag-recall relation becomes increasingly variable. A lag of 29, for example, can be realised only when the last test trial reproduces the answer at the first.

The estimated probabilities for yoked pairs have been increased nine-fold for the following reason: A yoked pair can be sourced from any previous answer that does not include the present cue (else the recall would be classified as an answer). An answer, on the other hand, can be sourced only from a previous trial on which the present cue was produced as a response. There are nine times as many trials that might be the source of a yoked pair as of an answer, but only the same number of opportunities for recall. Increasing the estimated probabilities of yoked pairs redresses this disparity. With this adjustment, yoked pairs show inverse recency up to lag 8 (Kendall rank correlation 0.500, *p* = 0.030, one-tailed, comparing lags 0–8 only); thereafter their probabilities decrease much as do those for answers. This replicates a similar result from Laming (2019). (Curiously, PS pairs also show inverse recency up to lag 4: Kendall rank correlation 0.800, *p* = 0.025, one-tailed).

The histogram bars at the right hand end of the abscissa in Fig. 5 indicate the proportions of, respectively, complete correct (BPS) recalls, correct BP, BS and PS pair recalls and yoked guesses (that is, sourced from a stimulus). The proportion of BPS recalls is calculated with respect to the total number (5880) of test trials; the proportions of correct pair recalls with respect to the residual numbers of trials (respectively, 2253, 2288, 2275) on which a correct pair recall might be observed (i.e., conditional on no BPS recall); and the proportion of yoked guesses again with respect to the residual number (2504; i.e., conditional on neither a BPS nor a correct pair recall). Excepting BPS recalls, the proportions of correct pairs and yoked guesses are comparable to the recall of cued pairs and yoked pairs at medium lag (say 15).

The black dashed curve is a reciprocal, agreeing with the proportion of answer recalls at lag 0 and approximating the variation of answer repetitions thereafter. It has equation

1 $$p\left( t \right) = 1/(2.09 + 0.65 \times {\text{lag}}).$$

The 2.09 is the reciprocal of the proportion of answers (lag 0), while the 0.65 is chosen to take the curve close to the greater part of the proportions of answer repetitions.

The inverse recency exhibited by yoked pairs requires explanation and poses the question how yoked pairs and erroneous answers are retrieved from memory in the first place. To summarise the argument in Laming (2019): The negative recency shown by yoked pairs means that they display a different lag-recency relation to cued pairs and answers. If yoked pairs were retrieved by selecting a component at random and using that component as cue to retrieve its partner, yoked pairs would exhibit the same lag-recency relation as cued pairs. So yoked pairs must be retrieved as pairs, from a single retrieval. Further, if repeated answers were recorded only when a yoked pair happened to match the trial cue, they would exhibit the same lag-recency relation as yoked pairs. So answers must be retrieved as triples.

Suppose the trial at Lag *l* does not contain a match to the trial cue. An answer retrieved spontaneously from that trial would tell the participant that that particular answer is wrong (because it fails to match the trial cue). Entering such a retrieval at the head of the mnemonic record would suppress a potential yoked guess (because from the head of the record the answer would be retrieved complete) and the positive recency of answers in Fig. 5 is thereby reflected in a negative recency of yoked guesses. It is assumed that, freed from this suppression, yoked pairs would exhibit the usual decreasing form of lag-recency curve. So, at a sufficiently long lag the probability of retrieving an answer decreases to the point that the suppression of yoked pairs is no longer apparent in relation to its own lag-recency curve. Hence the limited range of negative recency. In the case of PS pairs the explanation is simply that at short lags the complete answer tends to be retrieved in place of the pair.

### Lapse of time

The preceding analysis found that many recalls are repetitions, in whole or in part, of previous recalls. It follows that those previous recalls, most of them, must have been recorded in memory. Some of those previous recalls consisted simply of two unrelated guesses to which the participants in Lansdale and Laming (1995) accorded the lowest rating of confidence. It does not make sense to deliberately record such unimportant and irrelevant details; the simplest reading of these findings is that entry into memory, of whatever captures our attention, is automatic. This means that mnemonically a stimulus is no different from a recall and, from this point on, the distinction between stimuli and recalls, critical for the preceding analysis, is abandoned.

Instead, the analysis below traces each recall to its ultimate source, stimulus as well as answer, via a chain of intermediate recalls (cp. Fig. 4). Some of those recalls are recalls of sources created during the test session, but some can be shown to access a particular one of the original stimuli presented at the beginning of the experiment and accessed after some specific lapse of time. In this way recalls during test are sorted into some that are presumed to be recalls of previous recalls (though may alternatively have been retrieved directly from a stimulus) or guesses and others that must have accessed the original stimuli.

**Fig. 6:**
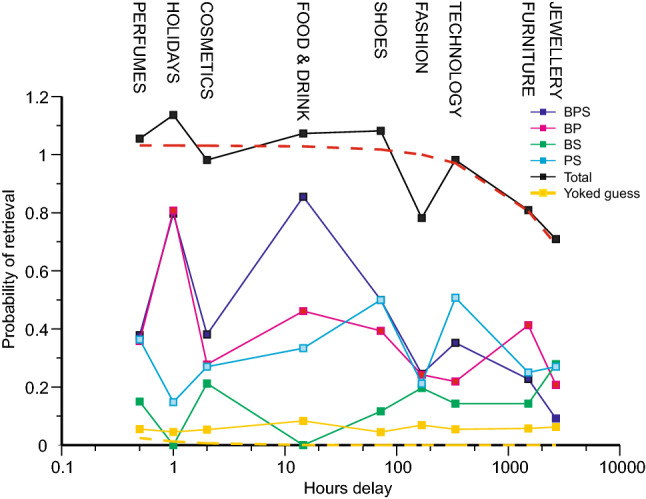
Proportions of retrievals from the original stimuli in relation to the lapse of time since presentation. The broken yellow curve continues the equation in Fig. 5; the broken red curve is a similar equation fit to total probability of recall

Extracting the data from those eleven participants who attended every testing session, Fig. 6 shows, for different categories of recall, the proportions of retrievals from original stimuli after each lapse of time. These proportions were calculated in this manner: For each category of recall in Fig. 6 there are trials on which such a recall, if it occurred, would have been sourced to the response on some previous test trial. Exclude all those trials. The data show the proportions of recalls (obtained directly from a stimulus) on the remaining test trials. ‘Total’ is simply the sum of all those proportions and in four cases exceeds 1. This happens when separate test trials independently access the same original stimulus, generating two different retrievals from the same source.

The broken yellow curve (overlying the abscissa) is a continuation of the hyperbola in Fig. 5, with the same parameter values. These (Eq. 1) were chosen to approximate the repetitions of previous answers, but at the same time approximated the retrievals of yoked pairs, excepting the initial range of negative recency. The yoked guesses in Fig. 6 are simply yoked pairs sourced from an original stimulus. Laming (2019) found that there was no difference between yoked pairs and yoked guesses, either in probability of retrieval, in confidence or in latency (but with the test trials there immediately following the presentation of the stimuli). Here there is a variable, sometimes long, delay between presentation of the stimuli and test, resulting in a much smaller proportion of yoked guesses.

The proportions of yoked guesses all lie above the curve, but this is because nearly all those yoked guesses are no more than apparent, arising from the chance coincidence of two separate individual guesses that happen to match one of the stimuli. When yoked guesses are aggregated together (Table 3), they are marginally significant; but the small samples represented here in Fig. 6 do not differ significantly from chance. It is plausible that this continuation of the hyperbola in Fig. 5 represents, approximately, the probability of retrieving a yoked guess directly from one of the stimuli.

The broken red curve in Fig. 6, on the other hand, has equation

2 $$p\left( t \right) = {1}/(0.{97} + 0.000{18}\times {\text{lapse of time in hours}})$$

and presents a passable account of the trend exhibited by the total probability of a recall of any kind. It needs to be emphasised that while these two relations, one for the retrieval of previous errors within the test series and the other for recall of stimuli after various, much longer, lapses of time are both are modelled with reciprocal functions, the constants in those functions (Eqs. 1 and 2) are very different.

‘Holidays’ and ‘Food and Drink’ show a much greater proportion of completely correct recalls than do the other product groups. To say that student participants are more interested in these products does no more than describe what is observed. A more substantial reason is suggested in the discussion.

### Discussion

The first analysis replicated many of the findings in Laming (2019)

1. Complete answers, cued pairs and yoked pairs are reproduced at highly significant frequencies. The only exception is BS pairs, either as cued or yoked pairs.
2. When one component of an answer chances to be the cue on the next trial, the probability of repeating that answer, complete, is high (0.835), except when the former answer is Null, that is, the confluence of two independent guesses.
3. Likewise, when the trial cue points to a previous complete recall of an answer (lag > 0), the probability of repetition is much increased, so that the probability of repetition of complete answers increases with successive repetitions, like completely correct recalls.
4. The probability of repeating a specific trial decreases with lag in the same manner as free recalls, except that yoked pairs show a negative recency up to lag 8.

All these results were reported by Laming (2019). There is nothing here about confidence, of course, because no confidence ratings were recorded; nor anything about latencies. But adding in those results from Laming (2019), leads to this simple model of what one might call the mechanics of memory:

1. Every event (a stimulus or a response or just a retrieval) to which the participant attends is separately recorded in memory, creating an ordered record of those events that have engaged the participant’s attention.
2. The compilation of the record is automatic; while attention to a stimulus is at the participant’s disposal, the consequent entry into memory is not.
3. The retrieval of a candidate response from memory is spontaneous; a retrieval becomes an overt response if it is compatible with the cue.

On the basis of this model the next important question concerns the functional decrease in accessibility with lapse of time.

### ‘Short-term memory’/‘Working memory’

The black dashed curve in Fig. 5 shows that up to 15 min the functional decrease is roughly reciprocal. Similar functions would work as well, because, although the data come from 5880 test trials, the incidences of repetition of errors are parcelled out into 150 sub-categories, containing many small samples. However, the relationship is certainly not 1/ln(time) and arguably not exp{−*t*} either.

Opportunities to study retention over 4 months are not common, but experiments testing retention up to 15 min are easy to arrange. Ricker, Vergauwe, and Cowan (2016) have published a spirited attempt to rehabilitate the idea of decay in memory proposed by Brown (1958). This idea has been controversial ever since, the chief alternative being forgetting due to interference, especially Keppel and Underwood (1962). That is an alternative because when participants fail to recall the stimulus, they do not say nothing, they give some other response instead. Without repeating the history (but see Ricker, Vergauwe, and Cowan, 2016), the nub of the controversy is as follows: When a stimulus item fails to be recalled, is that because it has decayed in memory beyond retrieval and some other response is then substituted in its place, or is the record of the stimulus still in good health, but some other item has overtaken it? This issue has been argued with experiments designed to favour one side or the other and usually requiring extra-experimental assumptions for the interpretation of their results. One such assumption is, of course, ‘decay’ and another such is ‘interference’.

Recently Sadeh, Ozubko, Winocur, & Moscovitch (2016) have argued that the distinction between interference and decay as agents of forgetting turns on how retention is tested, whether by ‘recollection’ or by ‘familiarity’, using the ‘remember/know’ procedure (Tulving, 1985). But this confounds the objective study of retention with the purely subjective distinction between recollection and familiarity.

The idea of spontaneous retrieval (Laming, 2019) leads to another and, to my mind, simpler view of this controversy. First, a stimulus is simply an entry in memory whose recall, or failure of recall, is recorded by the experimenter. Beyond attention from the experimenter, a stimulus is not mnemonically different from any other entry, a retrieval of an error or a guess. Next, if the item actually recalled is more recent (of smaller lag) than the stimulus, that is said to be ‘retroactive interference’; if it is more remote, ‘proactive interference’. It is not that the item recalled has ‘interfered’ with the stimulus; simply that the mechanics of memory has selected from several different sources according to their relative accessibilities (cf. Fig. 5). In an experiment designed to favour one side or the other in this controversy, participants might adopt some particular strategy that distorts the results. For example,

“Demonstrating the existence of time-based decay in short-term memory and pinpointing its rate is especially hard because our cognitive system has at least two mechanisms at its disposal to fight against forgetting: attentional refreshing and articulatory rehearsal (Camos, Lagner, & Barrouillet, 2011; Hudjetz & Oberauer, 2007)”. Ricker, Vergauwe, and Cowan (2016, p. 1989).

But attentional refreshing and articulatory rehearsal work by recording additional entries in memory; they do not alter the mechanics exemplified in Fig. 5. It needs to be emphasized that the recalls in Fig. 5 are incidental observations. The participants had no instruction about recalling their previous responses—nothing at all was said—those recalls happened naturally.

There has, of course, to be some decrease in accessibility like the black dashed curve in Fig. 5, because we are only able to recall one image at a time. The distribution of accessibility over a lifetime of memories cannot be uniform and it happens to be weighted towards the most recent. (What would human society be like if it were weighted at the other end?). Brown (1958) was taking a punt at the form of that relation. Cowan, Saults, and Nugent (1997) have suggested three different meanings of the term ‘decay’, and their first, “the loss of the ability to recall the target item across a period of time, not caused by interference.” is compatible with the reciprocal function in Fig. 5, and it would help to choose some term other than ‘decay’, which misleads. What matters is the nature of the relationship between lag and accessibility, and why it exists, and it would clear the air to eschew any term that purports a priori to dictate what that relation should be. Instead, what is needed is a hypothesis that relates directly to experimental observation. The Brown–Peterson experiment is one of the principal paradigms used to study retention of over short periods of time, and Laming (1992) showed all the principal findings could be accommodated with a weighting function decreasing as the reciprocal of elapsed time.

### Retention over the longer term.

The second analysis addresses retention over a longer time frame by identifying a category of recalls that necessarily (chance guesses excepted) accessed the original stimuli and thereby reflect the effect of delay. The trend of ‘total recalls’ can be modelled as a reciprocal (Eq. 2), like the relation in Fig. 4. But it is plain that the functional relation that approximates retrievals in Fig. 4 up to 15 min, does not apply to ‘total recalls’ in Fig. 5 for delays exceeding 20 min.

The most important deviation from that trend is the marked increase in complete BPS recalls after 1 h (relative to 30 min—supper was served in between these two test sessions) and, even more, after 14.6 h, overnight. The improvement after 1 h is reminiscent of Shepard’s (1967) study of the recall of pictures, a study that was specifically borne in mind in the design of this investigation. Shepard’s participants looked through an inspection series of 612 coloured pictures, followed immediately by a 2-alternative recognition test on 68 pairs of pictures, one seen before, one new. Four of the 34 participants were retested two hours later with 68 different pairs of pictures; 96.7% correct on the immediate test increased to 99.7% (one error by one participant only) after two hours. But this is to compare the performance of a particular four participants with the complete cohort of 34.

The second improvement overnight might suggest the effect of ‘sleeping on it’ (Wagner et al., 2004). In that study participants who slept overnight between training on a problem and test were significantly more successful than those who had stayed awake for an equivalent length of time, either overnight or within the same day. However, the comparison here is between different product groups, and it is plausible that Holidays (1 h) and Food and Drink (14.6 h) proved much more memorable to student participants than, say, Perfumes (30 min) and Jewellery (4 months). It was assumed, by default, that, provided care was taken to exclude extraneous cues to the associations between Brand, Picture and Slogan, the different product groups would be comparable. This is manifestly not so (and constitutes a caution for further research). It presents a problem that invites comparison with the work of Ballard (1913).

Ballard was an HM Inspector of Schools in London during the first decade of the twentieth century, a time when rote learning of poetry was a substantial component of primary education. He took advantage of his appointment to test the learning ability of many groups of schoolchildren, mostly aged 11–14. They would be asked to learn a poem with deliberately insufficient time to achieve complete mastery and were then asked to write out as much as they could remember. Ballard would return unexpectedly a few days later and ask the schoolchildren to write the poem out again. Scoring the number of whole lines reproduced without error, that number increased on repeated test, over the score on the initial test, by up to 20% (see Woodworth & Schlosberg, 1955, Fig. 25–6, p. 794). Since retention usually decreases with lapse of time, this increase seemed paradoxical, and was labelled ‘reminiscence’.

Reminiscence (more recently ‘hypermnesia’) has proved a fragile phenomenon, sometimes difficult to replicate (for reviews, see Payne, 1987; Erdelyi, 2010). But the principle that every event to which the participant attends is separately recorded in memory means that lines of poetry written during the initial test would have been recorded in memory and available for retrieval on a subsequent test. Moreover, because those lines had been *output*, they would have been more accessible than the original learning (cf. the ‘generation’ effect, Slamecka and Graf, 1978, and the ‘testing’ effect, Izawa, 1971, p. 201; Roediger and Karpicke, 2006). This mechanical manipulation of entries in memory was validated by Roediger and Thorpe (1978), who presented their participants with a list of 50 words or 50 pictures (to be recalled by verbal label). Participants asked to recall the list three times in successive 7 min periods produced a greater number of words in each successive period, presumably by retrieving their previous responses. However, their cumulative recall was no greater than that of a control group given 21 min for a single recall, suggesting that hypermnesia results simply from increased opportunity to recall.

It is relevant that Ballard scored the number of lines reproduced without error. This is analogous to selecting completely correct recalls only in Fig. 4. Ballard’s second test, of course, showed an increased amount recalled (as did Roediger and Thorpe, 1978, above). But the schoolchildren had no access to the poem in between whiles and the mnemonic process has to be a martingale. If Ballard had examined lines that were reproduced incorrectly, he surely would have found his school children repeating their errors as well on the second test. In short, reminiscence looks to be analogous to the increase in the proportion of completely correct recalls with successive tests in Fig. 4 and so an artefact resulting from a biased selection of data. Nevertheless, Ballard’s work has specific relevance to the enhanced recall of ‘Holidays’ and ‘Food and Drink’.

Ballard discovered that if his schoolchildren had previously learned the poem set for them to learn (but four years previously; Ballard, 1913, p. 35), reminiscence was greatly increased (147% in the space of two days). It is plausible that the student participants in the present experiment had previously looked at advertisements for Holidays and for Food and Drink to a much greater extent than, say, Perfumes or Jewellery. Analogy with Ballard’s report suggests a reason for enhanced recall.

### Experiments on retention

There is a practical difficulty to studying retention over long periods of time – commanding the attendance of a sufficiently large cohort of participants. This was a student project using friends of the experimenters – easily engaged, but their attendance was subject to other demands and had to be fitted in to their academic year. The entire cohort needed to be tested after each different lapse of time. Using different sets of material to test after each different delay finessed the problem of interference (confusion between different sets of material), but those different sets (product groups of advertisements) do not appear comparable. Shepard (1967) showed his participants the same inspection series of 612 coloured pictures, but then tested different sub-cohorts after each delay, testing each sub-cohort on the same test series. This finessed the problem of interference and also equated the material of the tests, but shifted the potential lack of comparability onto the sub-cohorts. Kruger (1929), using lists of 12 monosyllabic nouns, arranged for different participants to be tested on different lists after different delays in a Latin square design. This equalised both participants and material with respect to the different intervals of retention. The ideal solution, of course, is captive participants who can be tested over successive delays in succession (Ebbinghaus, 1964; Bachem, 1954). It avoids all these problems, but, assuming the present participants could have been persuaded, the present experiment would then have taken seven months.

### The fragmentation hypothesis

Very recently Joensen, Gaskell & Horner (2019) have posed the question whether a complex stimulus (a triple associate in their experiments) is forgotten all-or-none or by progressive loss of individual components. This is the same question with which this present investigation began. Their triple associates consisted of a location, a famous person and a common object, with 60 alternatives for each component that could be substituted in any combination. Each triple was presented as three pairs (location, person), (person, object) and (object, location) on separate trials, interspersed, of course, with the presentation of other pairs. It was assumed that the three separate pairs would be integrated into a single engram. Recall was tested by presenting one component as cue with a six-alternative forced-choice test for one other component, one component only, giving six separate test trials for each triple. Half the triples were tested immediately and the other half after 12 h (1 week in Experiments 2, 3 and 4).

Joensen et al.’s initial unit of analysis was a 2 × 2 table (Table 4 below) recording the combinations of responses to each cue. They calculated a measure of ‘Retrieval dependency’, which was the excess of the sum of the diagonal entries over what those entries would have been if accuracy were maintained but the responses were otherwise independent; that is

3 $${\text{Retrieval dependency}} = \left( {a + d} \right) - \left[ {\left( {a + b} \right)\left( {a + c} \right) + \left( {b + d} \right)\left( {c + d} \right)} \right],$$

where *a*, *b*, *c* and *d* are the proportions of entries in each cell in Table 4, so that *a* + *b* + *c* + *d* = 1.

**Table 4:** Initial unit of analysis in the experiment by Joensen, Gaskell & Horner (2019)

Cue A	B√	¬B
C√	*a*	*b*
¬C	*c*	*d*

The entries *a*, *b*, *c* and *d* are proportions, so that they sum to 1

“If complex events fragment as a function of forgetting, such that some aspects of the memory trace are forgotten more quickly than others…, then we would expect to see a decrease in dependency over time.” (Joensen et al., p. 12).

However, there is another, simpler and more informative, way to answer the question posed by Joensen et al. Assemble the responses to all three cues pointing to a given engram in a 3 × 3 matrix as in Table 1. Then the structure of the engram (cf. Fig. 1) dictates a particular combination of correct responses (see Jones, 1978; Lansdale & Laming, 1995). Conversely, the pattern of correct responses in a matrix like Table 1 enables the structure of the engram to be identified individually for each separate triple, whence the answer to the question posed by Joensen, Gaskell & Horner (2019) follows immediately.

However, there are problems. First, correct guesses will obscure the pattern of correct responses (and it is now a pity that Joensen et al. did not present their participants with a 60-alternative forced choice). Second, even after correcting for guessing, one must expect combinations of responses that do not fit any of the patterns in Fig. 1 (see Lansdale & Laming, 1995). Such patterns arise because many of the recalls are not recalls of the original engram, but of intermediate recalls from previous test trials (Laming, 2019, and the analysis above). Here is the crux of the problem: The analysis in Table 1 (and in Tables 4 and 5 and in Joensen, Gaskell & Horner, 2019) assumes that all cues pointing to a given triple access the *same* engram. This is not so here, nor in Lansdale & Laming (1995; see Laming 2019), and it is to be expected that if Joensen et al., performed a similar sequential analysis on their data, similar relationships would be discovered.

It seems intuitive that the projection of the engram of a triple associate onto recall should be one of the five patterns in Fig. 1 and, moreover, that forgetting must subsist either in the loss of individual elements or of the disappearance of the entire engram. It is intuitive because these are all the questions that one can ask of a 3-component engram.

But this is not at all necessary. Analysis of the recalls of the advertisements showed that many were retrievals of previous recalls (Fig. 3) and that such recalls occur in chains (recalls of recalls of recalls, Fig. 4). More generally, failure to recall a particular engram inserts an additional entry at the head of the mnemonic record, so that the probability of recall of the original engram on a second attempt is reduced – its lag is increased by 1 and this results in reduced accessibility (Fig. 5). One might suppose that, if the attempt to recall were continued long enough, it would eventually succeed, but this turns out not to be true – not even in an infinite series of attempts (Laming, 2009). So, forgetting – specifically, failure to recall—is consistent with complete preservation of the original engram. Moreover, because one can never know that distinct recalls are retrievals of the same engram, it is not even possible to identify its structure.

### How then does memory work?

This Discussion began with a simple model of what one might call the mechanics of memory:

Every event (a stimulus or a response or just a retrieval) to which the participant attends is separately recorded in memory, creating an ordered record of those events that have engaged the participant’s attention.

This is suggested here by the analysis of repeated errors. The total number of trials on which some previous error was repeated is 1495. Delete from the total number of test trials (5880) those on which recall was completely correct, and delete also the first test cycle through the stimuli, because an error cannot be repeated until after it has been made. This leaves 2272 trials of which 65.8% included a repetition of an error of some kind. We cannot, of course, know about those previous responses that were not repeated, but repetition of previous errors in recall is common.

The compilation of the record is automatic; while attention to a stimulus is at the participant’s disposal, the consequent entry into memory is not.

This may seem counter-intuitive. I draw this distinction: Fixing attention on particular stimulus material (e.g., nonsense syllables) may require a great deal of conscious effort or (a football match) may not. But attention then guarantees entry into memory; entry into memory is a pragmatic definition of attention. It enables all sorts of everyday events to be recorded without any specific intention to retain. Some of the response combinations repeated in Fig. 3 consisted of two independent guesses (Null responses). It is difficult to think of any other reason why such responses should be retained in memory for future recall.

The retrieval of a candidate response from memory is spontaneous; a retrieval becomes an overt response if it is compatible with the cue; that is, a cue functions only in retrospect to select an appropriate response after it has been retrieved.

Yoked pairs in Table 3 are an example. They are compatible with the trial cue, but otherwise have no connection with it.

In free recall, after a first flush of recalls accessed by continuing the process of rehearsal (Laming, 2006, 2008), recalls are found to include words already recalled, intrusions from previous lists and even from outside the experiment altogether (Diesfeldt, 2017; Howard & Kahana, 1999; Laming, 2012, reanalysing data from Murdock and Okada, 1970). The mechanics exemplified in Fig. 5 brings a word to mind: Is this one of the words I was asked to remember? Is it a word I have already recalled? How is the participant to know? Within the context of the experiment such a retrieval is spontaneous and the participant has to guess: Davis, Geller, Rizzuto, and Kahana (2008) have demonstrated similar intrusions in the recall of paired associates.

The analysis of repeated errors affords a more primitive insight into the mechanics of memory than other presently existing procedures. The serial position curve of free recall (Murdock, 1962; see Laming, 2010 for its variants) is well known; the data in Fig. 5 exemplify the process that generates that curve. Likewise, data that suggest all-or-none learning and the fragmentation hypothesis are generated by the repetition of previous recalls, correct and part-correct (Laming, 2019). Repetitions of previous errors are incidental observations, unconstrained. They are properties of memory, not of the experiment, and should extrapolate without restriction.

The relation between accessibility and lapse of time, however, presents an unsolved problem. The analysis of repeated errors provides data (Fig. 5) on the decrease in accessibility over the short term, up to 15 min. That same analysis identifies certain responses as retrievals from the original stimuli presented at various times previously. The total proportion of such retrievals (Fig. 6) provides data on accessibility over the longer term, 20 min to 4 months. Both relations can be characterised by a reciprocal function (Eqs. 1 and 2), but the constants of those functions are very different.

There have been very many studies of retention over short periods of time and a proportionate number of models, so much so that it has become common to speak of ‘short-term memory’ or ‘working memory’ as though these topics had no connection with retention over the longer term. But there is, to my knowledge, no experimental evidence to show that there are two distinct processes. The disconnect proceeds from the ready availability of participants for studies over a short term compared with the difficulty of testing memory over much longer intervals (autobiographical memory excepted, Bahrick 1983, 1984).

This present study has recorded recall over both the short term and also over much longer intervals under circumstances in which the only relevant variable appears to be lapse of time. The data show that accessibility of an original stimulus is absolutely less than that of a recent test trial (of course), but extrapolation of Eq. 2 to short delays would give an enhanced recall, greatly enhanced over that represented by Eq. 1 (cf. Figure 6). So, if there are two distinct processes, one for retrieval in the short term, up to 15 min, and the other over longer terms, why can the ‘longer term’ process not be brought to bear on short-term retrieval? Putting this question the other way round, how does the short-term process, as reflected in Fig. 5, produce such enhanced recall from the original stimuli? Is this not a comparison of like with like, after all? Or does the comparison with the report from Ballard (1913) hint at a much more complicated structure over the long term?

## Electronic supplementary material

Below is the link to the electronic supplementary material.

Supplementary file1 (DOCX 19 kb)

Supplementary file2 (XLS 42 kb)

## Data Availability

The part-processed data on which Figs. 3, 4, 5 and 6 are based are set out in Advert_Data.xls, which accompanies this submission as supplementary material. The raw data contain personal information, but other tables of part-processed data, analogous to those in Advert_Data.xls, may be available from the author on reasonable request.

## Footnotes

1 I thank Kate Paveley and Sally Wheatley who carried out this experimental project as part of their third-year coursework in the Department of Experimental Psychology, University of Cambridge, in 1983–1984.

2 This experiment was conducted in 1983–1984 with the resources and facilities then available.

3 It is possible for the same answer to match both a cued pair from one previous trial and a yoked pair from another, but not from the same trial. Selecting the most recent of multiple matches gives a unique categorization.

4 A sequence of random variables *X*_1_, *X*_2_,…,*X*_*n*_ is a martingale if the expectation of *X*_*n*+1_ = *X*_*n*_. An immediate consequence is that the variance of the variables *X*_*n*_ is continually increasing. Since the proportions in Fig. 3 are constrained to the interval (0,1), each sequence of probability of recall will ultimately diverge to either 0 or 1.
