# Supplementary material for: Recall of advertisements after various lapses of time
Source: Psychol Res. 2020 Sep 17;85(6):2428–43. doi: 10.1007/s00426-020-01408-y (PMC8357737; doi:10.1007/s00426-020-01408-y)
Supplement: Supplementary file 1 — Supplementary file1 (DOCX 19 kb) [file 426_2020_1408_MOESM1_ESM.docx]

The current study presents a conceptual replication of laming 2019 which was a reanalysis of data from previous research in cued recall, in which stimuli composed of various different combinations of 3 specific types of components -color, pattern & object- were presented to subjects, and then cued by one component participants’ were requested to recall the other two components (Landsdale & Laming, 1995). In this study analyzing data from an experiment in 1983-1984, in which subjects were presented magazine advertisements composed of various combinations of brand picture and slogan. In addition, in the current study the study addresses the effects of time on recall of response patterns, and the difference in result patterns between long-term and short-term memory. There were 9 different groups with varying intervals between advert encoding and testing, ranging from 20 minutes up to 16 weeks.

As in Laming 2019, the author focuses on the relation of current combinations to all previous combinations, as opposed to the relations with the 3 specific components (examined in the 1995 research). The analysis examines the errors committed as opposed to merely the accuracy of recalls, focusing mainly on the sources of these responses. The author proposes that results affirm the model set out in laming 2019 whereby errors may reflect participants’ tendency to recall their own previous responses. This claim is based on results of frequencies of response, conditional probabilities following certain responses and analysis of lag recall curves.

From the reanalysis the author draws three main conclusions: 1. Events are separately recorded in memory, in an ordered fashion. 2. All events which are attended to will be automatically encoded into memory, people may have control over attention but over automatic memory recording. 3. Retrieval is a spontaneous process of selection of candidates from memory, an overt response is formed if retrieval is compatible with a cue.

The analyses presented are compelling, and the issues discussed are very interesting. These issues relate substantially with renewed interest and research into forgetting, all-or-none forgetting and errors in recall. Overall, the research is valid and of interest, however, there are several important issues regarding wording, argument structuring, formatting and ease of read which I believe ought to be addressed before publishing the manuscript. In addition, I included a few theoretical points to ponder, and some literature on the subject the author may find useful.

- There have been several papers in recent years in topics related to those discussed in this manuscript which the author may wish to consider. Namely:

**Re. intrusions in recall:** Davis, O. C., Geller, A. S., Rizzuto, D. S., and Kahana, M. J. (2008). Temporal associative processes revealed by intrusions in paired-associate recall. Psychonomic Bulletin & Review, **15**(1), 64-69.

This is mentioned on p.40 in the context of intrusions in free recall, which are more widely known.

**Re. accessibility and forgetting:** Berens, S. C., Richards, B. A., & Horner, A. J. (2020). Dissociating memory accessibility and precision in forgetting. Nature Human Behaviour, 1-12.

I have not had a sight of this article, which appears to be still embargoed online. However, the abstract (PubMed) suggests a less precise formulation of the question already posed by Joensen, Gaskell & Horner (2019) and it is questionable whether it would add anything to the argument.

**Re. Interference Vs. Decay in memory:** Sadeh, T., Ozubko, J. D., Winocur, G., & Moscovitch, M. (2016). Forgetting patterns differentiate between two forms of memory representation. *Psychological science*, *27*(6), 810-820.

I comment on p. 30; but this study used the ‘remember/know’ procedure (Tulving, 1985) which, in my judgment, confounds the objective study of retention (by recall) with the purely subjective distinction between recollection and familiarity.

-It ought to be more plainly stated (more than a footnote), and at a sooner point in the paper that the experiment was conducted in 1983-1984.

Yes, this is now stated up front.

- The author might consider moving some of the points from the discussion to the introduction. Such as presenting the work of Joensen, Gaskell & Horner (2019).

Joensen, Gaskell & Horner (2019) is now mentioned up front as a recent exploration of the fragmentation hypothesis.

-Reordering the Introduction might help readability – Starting with the fragmentation hypothesis, explaining about the model of Laming 2019, and only then introducing the current study. In addition, it would better help understanding the article if there was further elaboration regarding the all-or-none studying model laid out in Laming 2019, and its relation to the fragmentation hypothesis. Such that readers may have enough of an understanding of the theoretical background without having to read Laming 2019.

I am very happy to go along with this suggestion.

The conclusions copied from Laming (2019) have now been delayed until the Discussion.

- The reasoning and discussion laid out in the sub chapter “Retention over longer term” are very difficult to follow. Perhaps it ought to be revised, with a more “on-point” claim, and less digression into (albeit interesting!) anecdotes about past research.

I have a problem in the greatly increased number of correct recalls of ‘Holidays’ and ‘Food and Drink’ relative to other product groups. This difference is now emphasized and that this difference requires explanation. This is how reminiscence becomes relevant. I have also deleted the long quotations in this sub-section, summarizing their import each in a sentence.

-It is unclear what the author wishes to claim regarding differences between long and short term memory and on which data he bases this. While there may indeed be an important claim here to be taken from this study it is currently stated cryptically in a few lines at the end of the abstract and the end of the discussion without proper explanation.

The final section has been rewritten to make these points

1. The conceptual distinction between studies of ‘short term memory’ or ‘working memory’ and long-term forgetting is not a matter of experimental demonstration, but simply a product of the great ease of conducting the first kind of study where the other is difficult.
2. The present study compares recall over both the short term and also over much longer intervals under circumstance in which the only relevant variable appears to be lapse of time.
3. Extrapolation of long-term accessibility to short intervals would make short term recall much better than it is. Alternatively, extrapolation of short-term accessibility to longer intervals poses the contrary problem.
4. This questions whether the comparison in this experiment between short and long term is truly like with like.

- It may be prudent to address the differences between the triad of components from Laming 2019 (Color, Pattern, Object), and that in the current study (Brand, Slogan, Picture). While it’s tempting to see in both cases simply three components compromising a complex event, there is possibly meaning to the different components chosen. Whereas in Laming 2019 the “Pattern” and “Color”, are cues related to the visuospatial context of the combination, in the current study “Brand” and “Slogan” are both semantic textual features. It is also worth further explaining the point made (Pgs. 16,28) that Brand and Slogan “do not cohere well”

This is now a matter of comment on pp.17/18.

- Regarding the Issue of Decay in memory, there have also been support for this claim from neurobiological studies which have shown evidence for active clearance via neurogenesis (Frankland, Köhler, & Josselyn, 2013) or dopaminergic “forgetting cells” leading to intrinsic forgetting (Davis & Zhong 2017).

If the idea is that memories decay as a *negative exponential*, the volumetric content of our memories would be constant. This seems to me so incompatible with human experience, to say nothing of experimental observation, that it is not, to my mind, worth consideration. If, on the other hand, ‘decay’ does not actually mean “negative exponential” (cf. Cowan, Saults, and Nugent (1997)), do not use the word ‘decay’.

- Regarding the author’s proposed claim of retrieval as a spontaneous process (Pg. 29, 40). According to Views which see recall as a result of contextual dynamics (see healey long & kahana, 2019), retrieval is based on similarity of the internal neural context during the time of retrieval to that during the time of study. This account also has some support from imaging studies showing a connection between encoding-test similarity and memory performanced (Danker, Tompary, & Davachi, 2017). Thus, although it may be useful to assume spontaneity for modelling purposes it is likely that retrieval might not be purely spontaneous but rather dependent on the flow of the multidimensional internal context.

This is a very valid point that I choose not to debate in an already long paper. Yoked pairs and guesses are categories of recall that patently do not depend on the agency of a cue. It is simplest to propose that all retrievals in this experiment are spontaneous and are output if they are compatible with the trial cue. So, ‘spontaneity’ is relative to retrieval in this experiment.

But this leaves the question how a particular response combination, or a word in a free recall experiment, comes to mind in the first place. I do not offer any answer to that question beyond the statistics displayed in my Figure 5.

You might think that the healey long & kahana, 2019 provides an answer. But that paper perpetuates an error in interpretation, dating back to Kahana (1996), that it would be quite out of order to debate here. Suffice to say that I can suggest, and justify, a much simpler ‘contextual dynamics’ than is presented here.

- In regard to the claim that all events which have had attention “paid” to them will be recorded similarly in memory (Pg. 29, 40). Recently there has been a renewed interest in the study of forgetting alongside that of memory, which has shown forgetting not merely to be the absence of successful retrieval but also an active (and perhaps adaptive) process. Behavioral paradigms as think/no-think (Anderson & Green, 2001), have displayed such malleable control, and indeed the biological findings in support of active decay mentioned above, thus (though again for parsimonious modelling purposes it may suffice) to get a whole picture of the processes underlying forgetting it may not be enough to simply explain them in terms of attention.

Anderson & Green’s participants underwent training to suppress responding to a randomly selected set of word pairs. What did that training consist of? Of further trials recorded in memory, moreover because those training trials were more recent, they would have enjoyed preferential access over the original word pairs. “Impaired memory for suppression items indicates that there may be an executive control process that suppresses (reduces activation of) the unwanted memory itself” – the executive control process subsists, I suggest, in more recent entries to memory that enjoy preferential access. The same process works in therapy to overcome traumatic experiences.

There is a subtle unspoken issue here. Anderson & Green suppose, without spelling it out, that each experimental word-pair is recorded in a specific engram, whose retrieval can be suppressed by suppression training. But an alternative view is that Anderson & Green’s participants are actually repeating their training responses, as the participants did in the experiment analyzed here.

I think this distinction is too remote from the present argument to justify comment.

- In addition to the proportions of responses compared to the expected “chance”, it may be helpful to perform a bootstrapping analysis and add to Table 2 bootstrapped Confidence intervals (e.g. 95 %) in order to strengthen the claim that recall frequencies do indeed differ from chance patterns.

In Table 3 I have added 95% confidence intervals next to the numbers observed. These intervals have been calculated directly from the generalized binomial distributions used for the significance tests. These calculation assume that individual repetitions are statistically independent, which is not quite correct. Each individual repetition may provide, or deny, an opportunity for a further repetition at some subsequent trial. But I think this is the best; it is certainly the simplest.

Bootstrapping requires re-sampling from independent observations and the smallest, statistically independent unit of data here is a complete sequence of 30 trials from a single participant. But such a re-sampling would fail to address the point at issue, which concerns the frequencies of repetitions *within* such a sequence.

- Table 3 and figure 3 are somewhat repetitive. One may be enough.

The original Table 3 has been deleted.

**References**

Anderson, M. C., & Green, C. (2001). Suppressing unwanted memories by executive control. *Nature*, *410*(6826), 366-369.

Danker, J. F., Tompary, A., & Davachi, L. (2017). Trial-by-trial hippocampal encoding activation predicts the fidelity of cortical reinstatement during subsequent retrieval. *Cerebral Cortex*, *27*(7), 3515-3524.

Davis, R. L., & Zhong, Y. (2017). The biology of forgetting—a perspective. *Neuron*, *95*(3), 490-503.

Frankland, P. W., Köhler, S., & Josselyn, S. A. (2013). Hippocampal neurogenesis and forgetting. *Trends in neurosciences*, *36*(9), 497-503.

Healey, M. K., Long, N. M., & Kahana, M. J. (2019). Contiguity in episodic memory. Psychonomic bulletin & review, 26(3), 699-720.
